# Sialic acid transport and catabolism are cooperatively regulated by SiaR and CRP in nontypeable *Haemophilus influenzae*

**DOI:** 10.1186/1471-2180-10-240

**Published:** 2010-09-15

**Authors:** Jason W Johnston, Haider Shamsulddin, Anne-Frances Miller, Michael A Apicella

**Affiliations:** 1 Department of Microbiology, Immunology, and Molecular Genetics, The University of Kentucky, Lexington, KY, USA; 2 Department of Chemistry, The University of Kentucky, Lexington, KY, USA; 3 Department of Microbiology, The University of Iowa, Iowa City, IA, USA

## Abstract

**Background:**

The transport and catabolism of sialic acid, a critical virulence factor for nontypeable *Haemophilus influenzae*, is regulated by two transcription factors, SiaR and CRP.

**Results:**

Using a mutagenesis approach, glucosamine-6-phosphate (GlcN-6P) was identified as a co-activator for SiaR. Evidence for the cooperative regulation of both the sialic acid catabolic and transport operons suggested that cooperativity between SiaR and CRP is required for regulation. cAMP was unable to influence the expression of the catabolic operon in the absence of SiaR but was able to induce catabolic operon expression when both SiaR and GlcN-6P were present. Alteration of helical phasing supported this observation by uncoupling SiaR and CRP regulation. The insertion of one half-turn of DNA between the SiaR and CRP operators resulted in the loss of SiaR-mediated repression of the transport operon while eliminating cAMP-dependent induction of the catabolic operon when GlcN-6P was present. SiaR and CRP were found to bind to their respective operators simultaneously and GlcN-6P altered the interaction of SiaR with its operator.

**Conclusions:**

These results suggest multiple novel features for the regulation of these two adjacent operons. SiaR functions as both a repressor and an activator and SiaR and CRP interact to regulate both operons from a single set of operators.

## Background

Sialic acid (5-*N*-acetylneuraminic acid, Neu5Ac) is used by nontypeable *Haemophilus influenzae *(NTHi) to assist in the evasion of the host innate immune response. Sialic acid is used to decorate the cell surface, primarily as the terminal non-reducing sugar on the lipooligosaccharride (LOS) and the biofilm matrix [1,2]. The presence of sialic acid on the cell surface protects the cell from complement-mediated killing, although the precise mechanism of this protection is unknown and may even vary among strains of NTHi [3-5]. Regardless, the acquisition and utilization of sialic acid is a crucial factor in the virulence of the majority of NTHi [3,4,6-8].

**Figure 1:**
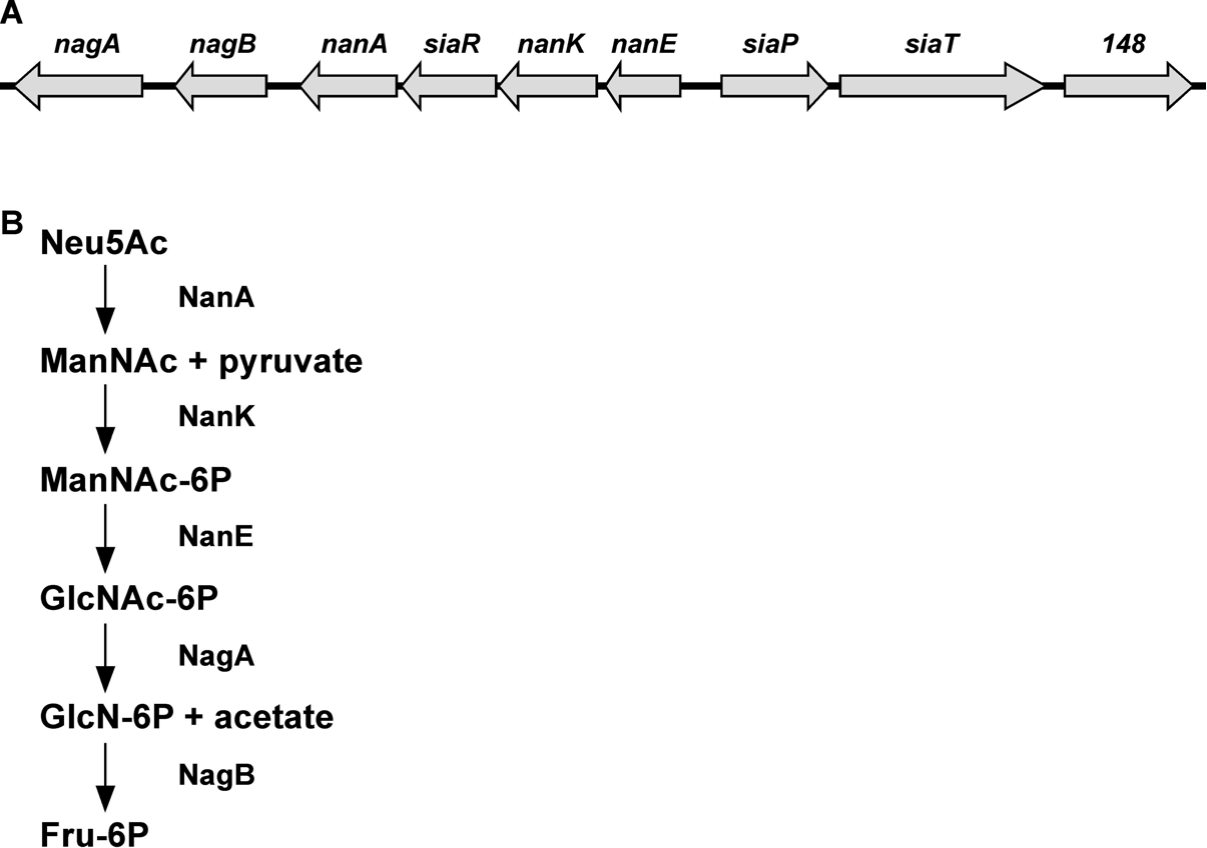
**The sialic acid catabolic and transport operons and pathway**. A. Schematic diagram of the *nan *and *siaPT *operons. The *nan *operon encodes for the entire catabolic pathway and the transcriptional regulator SiaR. The *siaPT *operon encodes for the sialic acid transporter and YjhT, a sialic acid mutarotase. The accession numbers for the KW-20 Rd sequence are indicated below each gene. B. The sialic acid catabolic pathway.

NTHi cannot synthesize sialic acid and therefore must scavenge it from the host. NTHi possess a high-affinity transporter for sialic acid, encoded by *siaPT *(also referred to as *siaPQM*) [6,9,10]. The SiaPT transporter is a member of the TRAP transporter family, with SiaP functioning as the solute-binding protein and SiaT functioning as the transmembrane transporter protein. An ortholog of the *E. coli *sialic acid mutarotase *nanM *is found downstream of the *siaPT *operon (HI0148) [11], although *nanM *does not appear to be co-transcribed with *siaPT *in *H. influenzae *strain Rd [12]. The genes required for the catabolism of sialic acid are found in the adjacent, divergently transcribed *nan *operon (Figure 1A). The genes of the *nan *operon encode all the enzymes required to convert sialic acid to fructose-6-phosphate (Figure 1B), which can then enter the glycolysis pathway [13]. Prior to the decoration of the cell surface, sialic acid must be activated by SiaB, the CMP-sialic acid synthetase, forming the nucleotide sugar donor used by sialyltransferases [4]. Once transported into the cell, sialic acid is either catabolized by the enzymes of the *nan *operon or activated by SiaB. Thus, these two pathways compete for the same substrate [13]. The organism must therefore maintain a balance between these two pathways, ensuring that a sufficient amount of sialic acid is available to decorate the cell surface and adequately protect the cell from the host immune response.

Also present in the *nan *operon is the transcriptional regulator SiaR. SiaR is involved in the regulation of the *nan *and *siaPT *operons [14]. SiaR was found to repress the expression of both the *siaPT *and *nan *operons, thus regulating both transport and catabolism. Binding of SiaR to the intergenic region between these two operons was demonstrated and the region of DNA protected by SiaR was identified. As expected, it was found that inactivation of *siaR *lead to a reduction in surface sialylation, demonstrating the need to control the expression of sialic acid catabolism. In addition to SiaR, the cAMP receptor protein (CRP) was identified as a regulator of the *siaPT *operon, however a role in the regulation of the *nan *operon was not observed [12,14]. This is in part consistent with the observation that sialic acid is a cAMP-independent sugar [15]. In *H. influenzae*, CRP has been shown to regulate utilization of galactose, ribose, xylose, and fucose [15], in addition to regulating the development of competence [16].

We now report on the role of intermediates in the Neu5Ac catabolic pathway in SiaR-mediated regulation. Also, the potential interaction between SiaR and CRP was investigated. SiaR was found to utilize glucosamine-6-phosphate (GlcN-6P) as a co-activator in the presence of the CRP-cAMP complex. SiaR and CRP were found to act in a cooperative manner to regulate the expression of the divergent transporter and catabolic operons. Our results reveal a unique mechanism of regulation of two divergent operons regulated by two transcription factors from a single location.

## Results

### Promoter structure of the *nan *and *siaPT *operons

**Figure 2:**
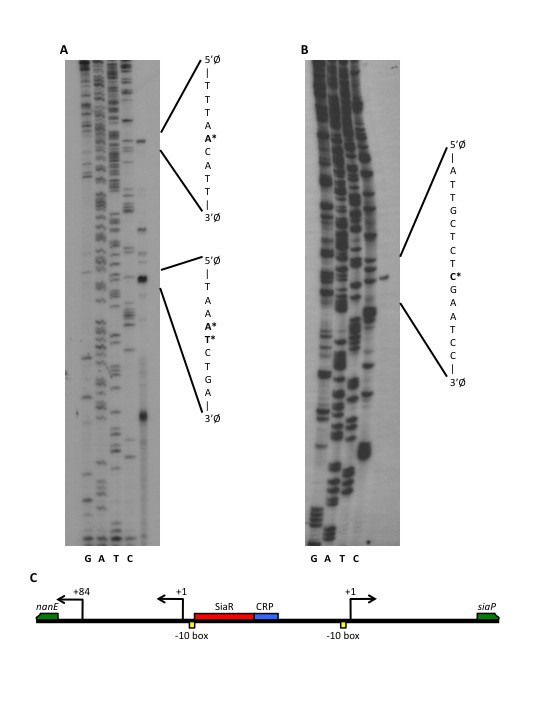
**Primer extension analysis of the *nan *and *siaPT *operons**. Complement of sequence surrounding the transcriptional start sites included with the sites indicated in bold and with asterisks. A. Primer extension analysis identified at least two major transcriptional start sites for the *nan *operon. Two bands were present for TS-2_*nan *_as indicated. B. Primer extension identified one start site for the *siaPT *operon. C. Schematic diagram of the *nan *and *siaPT *promoters. Binding sites for SiaR (red box) and CRP (blue box) are indicated as well as putative -10 boxes for TS-1_*nan *_and TS-1_*siaPT *_(yellow boxes).

The transcriptional start sites of the *nan *and *siaPT *operons were identified using primer extension analysis. Primers that bound in the *nanE *and *siaP *open reading frames were used. Two major start sites were identified for the *nan *operon, 104 (TS-1_*nan*_) and 20 (TS-2_*nan*_) bp from the start codon of *nanE *(Figure 2A). The presence of additional minor bands may be the result of addional start sites or RNA degradation or processing. The analysis identified a single transcriptional start site (TS-1_*siaPT*_) 107 bp upstream of the start codon of *siaP *(Figure 2B). This organization leaves 140 bp in between TS-1_*nan *_and TS-1_*siaPT*_. The putative CRP binding site is located at -59 to -80 relative to TS-1_*siaPT *_and at -59 to -80 relative to TS-1_*nan *_(Figure 2C). This organization suggests that the *siaPT *promoter falls into the class I group of CRP-dependent promoters [17]. A consensus -10 sequence was identified for TS-1_*nan *_and was found to partially overlap the SiaR binding site, consistent with the role SiaR plays in repression of the *nan *operon. The relative location of TS-1_*nan *_to the SiaR operator, in addition to the identification of a consensus -10 box, suggests that this start site would be primarily involved in SiaR-mediated regulation, however, the relative contribution of the two *nan *promoters will need to be examined in more detail.

### Glucosamine-6-phosphate is a co-activator for SiaR

Previous studies found limited activation of SiaR-regulated operons by sialic acid [14]. The potential for intermediates in the sialic acid catabolic pathway to influence regulation by SiaR was explored. *H. influenzae *is unable to transport any of the intermediate sugars or phosphosugars of the sialic acid catabolic pathway [13,18], therefore a mutagenesis strategy was necessary. Each gene encoding an enzyme in the catabolic pathway was deleted in an adenylate cyclase (*cyaA*) mutant strain, resulting in a series of double mutants. The Δ*cyaA *mutant strain was used to allow for CRP to be activated only by the addition of cAMP in subsequent experiments. In each mutant, sialic acid can be catabolized, but the sugar or phosphosugar immediately upstream of the inactivated enzyme should accumulate (Figure 1B).

The mutants were grown to early exponential phase and then either sialic acid, cAMP, or both were added. Expression levels of *nanE *and *siaP*, the first genes of the catabolic and transport operons, respectively, were compared using real time quantitative RT-PCR (qRT-PCR). RNA from a culture that received neither sialic acid nor cAMP served as a reference for each experiment. When both sialic acid and cAMP were added to cultures, expression of *nanE *was only moderately affected in strains 2019Δ*cyaA*, 2019Δ*cyaA *Δ*nanK*, 2019Δ*cyaA *Δ*nanA*, and 2019Δ*cyaA *Δ*nagA *(0.7- to 5-fold change). The most striking change in *nanE *expression occurred in 2019Δ*cyaA *Δ*nagB*, with expression elevated 83-fold (Fig, 3). This mutant would be unable to convert GlcN-6P to fructose-6P, thus accumulating GlcN-6P. These results suggest that GlcN-6P is a major co-activator in SiaR-mediated regulation.

**Figure 3:**
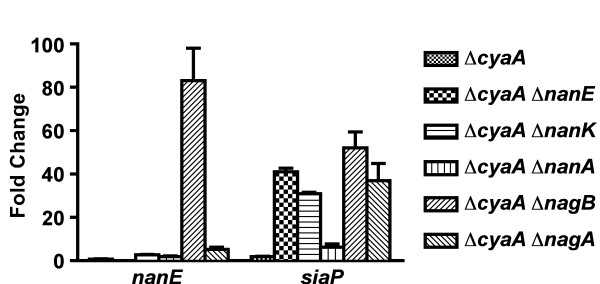
**Impact of metabolic impairment on the expression of *nanE *and *siaP***. 2019Δ*cyaA *and derived double mutants were grown in sRPMI. Sialic acid and cAMP were added 30 min prior to RNA extraction. Expression of *nanE *and *siaP *were measured by qRT-PCR. Results are presented as fold change relative to a culture that received neither sialic acid nor cAMP.

The regulation of *siaP *appears to be more complex. Expression of *siaP *was elevated 30- to 52-fold in strains 2019Δ*cyaA *Δ*nanE*, 2019Δ*cyaA *Δ*nanK*, 2019Δ*cyaA *Δ*nagB*, and 2019Δ*cyaA *Δ*nagA *(Figure 3). In contrast, increases of only 2- and 6-fold were observed in 2019Δ*cyaA *and 2019Δ*cyaA *Δ*nanA*, respectively (Figure 3). While SiaR can repress *siaP *expression [14], transcription of the transporter operon is more directly influenced by CRP. Despite this, *siaP *expression was not as responsive to cAMP in 2019Δ*cyaA *and 2019Δ*cyaA *Δ*nanA*. These results indicate that in these strains, SiaR is able to exert some control over *siaP *expression, however the mechanism in which this is accomplished is unclear.

### SiaR and CRP interact to regulate the adjacent *nan *and *siaPT *operons

**Figure 4:**
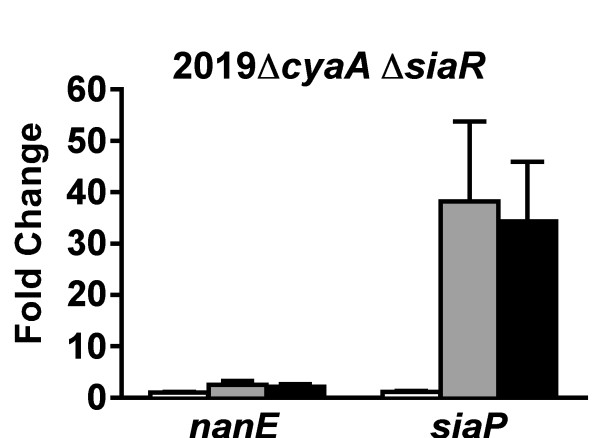
**Expression of *nanE *and *siaP *in 2019Δ*cyaA *ΔsiaR**. Cultures grown with sialic acid (open bars), cAMP (gray bars), and both sialic acid and cAMP (black bars) were compared to a reference culture that received neither.

Previous work demonstrated that in a *siaR *mutant, CRP and cAMP are unable to influence *nan *operon expression [14]. Since the current studies were performed using different mutant constructs, the experiments were repeated with the double deletion mutant to confirm the previously observed phenotype. The 2019Δ*cyaA *Δ*siaR *mutant was examined by qRT-PCR (Figure 4) and regardless of whether sialic acid or cAMP was added, expression of *nanE *did not change relative to the control. In the absence of SiaR, cAMP activated the expression of the *siaPT *operon, while the *nan *operon was unaffected.

**Figure 5:**
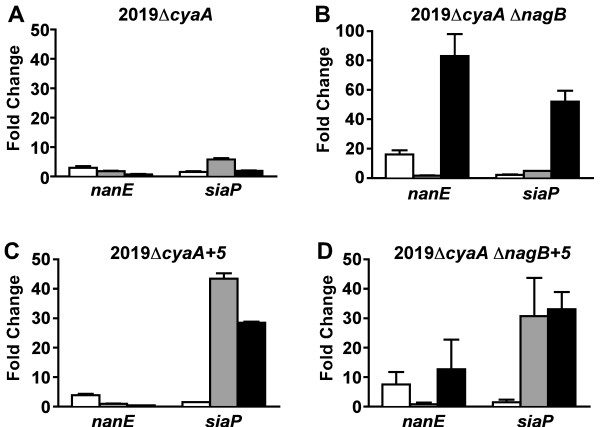
**Expression of *nanE *and *siaP *is altered by helical phasing**. Expression of *nanE *and *siaP *in 2019Δ*cyaA *(A), 2019Δ*cyaA *Δ*nagB *(B), 2019Δ*cyaA*+5 bp (C), and 2019Δ*cyaA *Δ*nagB*+5 bp (D). Cultures grown with sialic acid (open bars), cAMP (gray bars), and both sialic acid and cAMP (black bars) were compared to a reference culture that received neither.

Examination of the results obtained from 2019Δ*cyaA *Δ*nagB *revealed a large change in the expression of *nanE *that was cAMP-dependent (Figure 5B). The addition of sialic acid alone (which would be converted to GlcN-6P) led to a 16-fold induction of *nanE *while the addition of cAMP alone had no effect. The addition of both sialic acid and cAMP resulted in an 83-fold induction of *nanE*, indicating that the combination of GlcN-6P and cAMP significantly increase the induction of the *nan *operon. These results provide evidence of cAMP-dependent activation of both the *nan *and *siaPT *operons. Since cAMP does not induce *nanE *expression in a *siaR *mutant, this suggests that cAMP-dependent activation of *nanE *requires SiaR. SiaR and CRP may physically interact to activate *nan *operon expression.

To demonstrate that SiaR and CRP interact to regulate the *nan *and *siaPT *operons, alteration of helical phasing was used. Alteration of helical phasing is accomplished by the insertion of one half turn to the helix between the SiaR and CRP operators. Briefly, 5 bp was inserted between the SiaR and CRP binding sites in strains 2019Δ*cyaA *and 2019Δ*cyaA *Δ*nagB*, resulting in strains 2019Δ*cyaA*+5 and 2019Δ*cyaA *Δ*nagB*+5, respectively. These strains were examined by qRT-PCR and the results were compared with those obtained from the parent strains.

In the 2019Δ*cyaA *Δ*nagB *background, altered helical phasing resulted in a steep reduction in *nanE *expression (from 83-fold in 2019Δ*cyaA *Δ*nagB *to 13-fold in 2019Δ*cyaA *Δ*nagB*+5) in the presence of both sialic acid and cAMP (Figures 5B and 5D). The induction level of *nanE *in the presence of sialic acid and cAMP was similar to the expression observed when sialic acid alone was added. The 5 bp insertion eliminated the cAMP-dependent activation of *nanE *that was observed in the 2019Δ*cyaA *Δ*nagB *strain.

In both the 2019Δ*cyaA *and 2019Δ*cyaA *Δ*nagB *backgrounds, altered helical phasing also resulted in the induction of *siaP *when cAMP was added (Figures 5A and 5C). In the 2019Δ*cyaA*+5 strain, the 5 bp insertion led to a 43-fold increase in *siaP *expression in the presence of cAMP (from 6-fold in 2019Δ*cyaA*) and a 29-fold increase (from 2-fold in 2019Δ*cyaA*) when both cAMP and sialic acid were present.

Taken together, these results indicate that altering the helical phasing succeeded in uncoupling SiaR- and CRP-mediated regulation of the *nan *and *siaPT *operons. It resulted in *nanE *expression becoming unresponsive to cAMP, much like it is in the 2019Δ*cyaA *Δ*siaR *mutant. Altered helical phasing also prevented SiaR from exerting a negative influence on the expression of *siaP*. We conclude that the insertion eliminated the ability of SiaR and CRP to interact to regulate both the *nan *and *siaPT *operons.

### SiaR and CRP bind to their respective operators simultaneously

**Figure 6:**
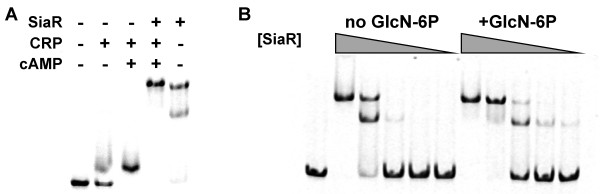
**Electrophoretic mobility shift assay**. A. Binding of both SiaR and CRP to the *nan*-*siaPT *intergenic region. Both SiaR and CRP bind to the probe individually and CRP binding is dependent on the presence of cAMP. Both proteins bind the probe simultaneously as indicated by the higher shift of the probe when both proteins are added. B. GlcN-6P enhances binding of SiaR. Two-fold serial dilutions of SiaR were added to binding reactions in the absence and presence of 100 μM GlcN-6P. More probe was shifted when GlcN-6P was present.

Binding of SiaR to an operator in the intergenic region between *nanE *and *siaP *was demonstrated previously [14]. The putative operator of CRP was identified *in silico *and was found to overlap the region protected by SiaR in a DNase I protection assay by three base pairs. The ability of both proteins to bind to their operators was examined using the electrophoretic mobility shift assay (EMSA). Both proteins were able to bind to a probe comprising the region between the two operons and CRP binding was dependent on the addition of cAMP (Figure 6A). When both proteins were included in the binding reaction, the DNA probe was shifted slightly higher than the SiaR-bound probe. This indicates that both proteins bind to their operators simultaneously, further supporting the hypothesis that the two regulators interact to regulate the adjacent *nan *and *siaPT *operons.

### GlcN-6P alters binding of SiaR to its operator

Many transcriptional regulators exhibit altered binding affinity for their operator sequences when a co-regulator is bound. To determine the effect of GlcN-6P on SiaR binding, EMSA was used. Serial dilutions of SiaR were incubated with DNA probes in the absence and presence of GlcN-6P (Figure 6B). In the presence of GlcN-6P, SiaR bound the probe and GlcN-6P slightly increased the binding affinity. While the presence of GlcN-6P did not result in a major change in the binding affinity of SiaR, the change in the shift does suggest that GlcN-6P is interacting with SiaR and impacting its ability to bind to its operator. Other phosphosugars of the sialic acid catabolic pathway (sialic acid, ManNAc, and GlcNAc-6P) nor GlcN-1P altered SiaR-binding (unpublished data) [14]. Taken together with the expression data, this demonstrates that GlcN-6P interacts with SiaR and has an effect on its DNA-binding properties. SiaR is not displaced from the DNA, but instead functions as an activator with GlcN-6P as a co-activator.

As in our previous studies [14], the binding of SiaR to the EMSA probe resulted in the appearance of two shifted bands (Figure 6). This was even more apparent when lower concentrations of SiaR were present in the binding reaction. The double shift is possibly caused by the binding of multiple SiaR proteins to the probe. This is a likely explanation, considering that the region protected by SiaR is large (53 bp) [14]. Further work will be necessary to determine the exact cause for the double shift.

### GlcN-6P accumulates in a *nagB *mutant

**Figure 7:**
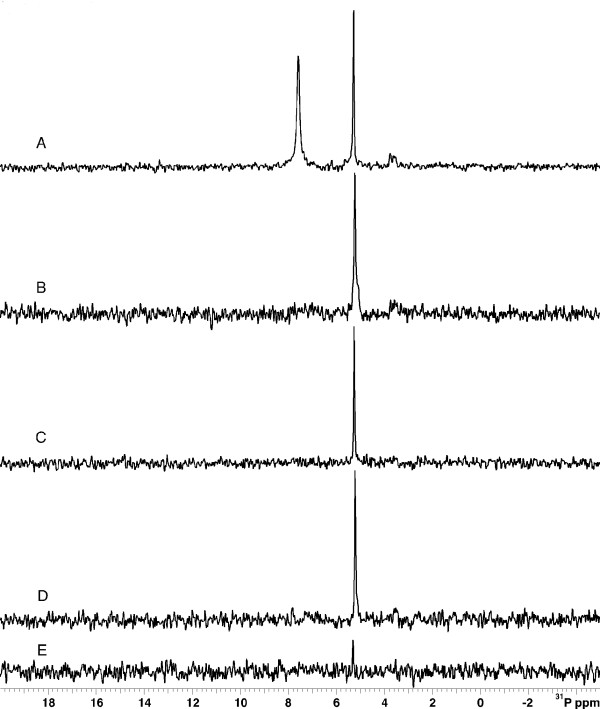
**Detection of intracellular GlcN-6P by **^**31**^**P NMR spectroscopy**. ^31^P NMR spectra were obtained following the growth of cells in the presence of exogenous cAMP and/or Neu5Ac. A. 2019Δ*cyaA *Δ*nagB *with Neu5Ac and cAMP. B. 2019 wild-type with Neu5Ac and cAMP. C. 2019Δ*cyaA *Δ*nagB *with cAMP. D. 2019 wild-type with cAMP. E. 2019 wild-type without supplement.

To confirm that Neu5Ac was transported and catabolized in the 2019Δ*cyaA *Δ*nagB *mutant strain, ^31^P NMR spectroscopy of intact cells was used. Cultures of wild-type 2019 and 2019Δ*cyaA *Δ*nagB *were grown to early exponential phase and cAMP and/or Neu5Ac were added and the ^31^P spectrum was obtained (Figure 7). A peak was detected near 5 ppm when cAMP was added to either strain. When Neu5Ac was added, a peak was detected near 7 ppm in the 2019Δ*cyaA *Δ*nagB *mutant that was absent in the wild-type strain. This peak was also absent in either strain when Neu5Ac was omitted. This indicated the accumulation of a significant amount of a phosphorylated compound in the mutant strain when exogenous Neu5Ac was present. Since the Neu5Ac catabolic pathway is blocked at NagB in the mutant strain, Neu5Ac would be converted to GlcN-6P, but not Fru-6P. Taken together with the interaction of GlcN-6P with purified SiaR, this indicates that GlcN-6P is accumulating in the 2019Δ*cyaA *Δ*nagB *mutant and is responsible for the activation of the *nan *operon.

## Discussion

The importance of sialic acid in the protection of NTHi from the host immune response requires that most of the sialic acid transported into the cell is activated by SiaB and utilized for the decoration of the LOS and biofilm matrix. Therefore, it is important that the catabolism of sialic acid be tightly regulated. We previously identified SiaR as a repressor for these two operons, in addition to the role of CRP in activating the expression of the transporter [14]. In this study, we present data that expands on our previous work, providing key details about the unique regulation of these adjacent operons. The two operons required for the transport and catabolism of sialic acid were found to be simultaneously regulated by SiaR and CRP in a novel mechanism for cooperative regulation. SiaR functions as both a repressor and activator, utilizes GlcN-6P as a co-activator, and interacts with CRP to regulate two adjacent and divergently transcribed promoters.

Since *H. influenzae *cannot transport the intermediates of the sialic acid catabolic pathway [13,18], mutants in each gene of the pathway were used to examine the role of the sugar and phosphosugar intermediates in the expression of the SiaR-regulated operons. Increased expression of the *nan *operon in the 2019Δ*cyaA *Δ*nagB *double mutant suggested that GlcN-6P functions as a co-activator. This is unusual because catabolic pathways are typically regulated by the presence of the substrate. SiaR likely uses GlcN-6P as a co-activator because sialic acid is utilized rapidly after transport by *H. influenzae*, either by activation with SiaB or catabolism beginning with NanA. Thus, sialic acid never accumulates to levels that would allow for sufficient expression of the transporter. In contrast, using GlcN-6P allows for moderate activation of *siaPT *to provide for transport of sialic acid. Since GlcN-6P can also be synthesized by the cell, expression of the transporter is not reliant on the presence of high levels of sialic acid, while increased sialic acid and catabolism will elevate levels of GlcN-6P and increase expression of the *nan *and *siaPT *operons. Even though GlcN-6P is not an endpoint in the catabolic pathway, transient levels of the phosphosugar likely allow for sufficient expression of the two operons.

**Figure 8:**
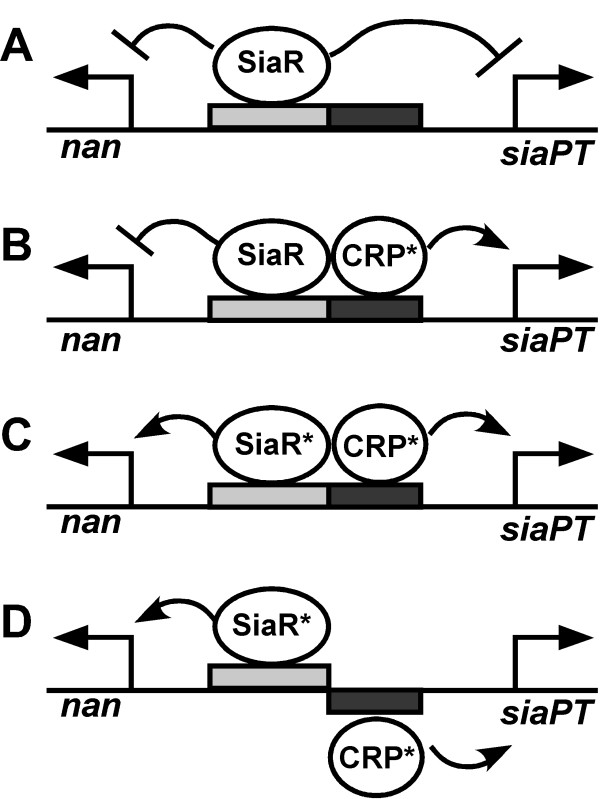
**Model of SiaR and CRP regulation of the *nan *and *siaPT *operons**. A. In the absence of sialic acid and cAMP, SiaR is bound to its operator and expression of the *nan *and *siaPT *operons is repressed. B. When cAMP is present, CRP binds to its operator and is able to activate the *siaPT *operon, but not the *nan *operon. C. When both GlcN-6P and cAMP are present, SiaR and CRP are active and interact to form a complex that activates both the *nan *and *siaPT *operons. D. In helical phasing experiments, insertion of one half-turn in between the SiaR and CRP operators prevents the regulators from interacting and thus maximal activation of the *nan *operon is not achieved.

In addition to identifying GlcN-6P as a co-activator, we found that SiaR and CRP interact to regulate both the *nan *and *siaPT *operons. Both regulators were able to bind to their operators simultaneously, demonstrating that binding of one protein does not prevent the binding of the other. cAMP-dependent activation of *nanE *requires SiaR. Furthermore, regulation of the two operons was uncoupled by the insertion of one half-turn of DNA between the SiaR and CRP operators. This insertion resulted in the loss of SiaR influence on *siaPT *expression and the loss of *nan *induction by cAMP. Based on this data and the proximity of the two operators, it can be concluded that SiaR and CRP interact to impact the expression of the two operons. This interaction may be the result of direct contacts between the two regulators or cooperative effects on DNA topography, however we cannot make any conclusions on the mechanism at this time. Our results have led to the description of a novel regulatory mechanism for two regulators of adjacent operons. This model, in the context of the experiments carried out in this study, is displayed in Figure 8. SiaR by itself functions as a repressor of both the *nan *and *siaPT *operons (Figure 8A). When cAMP levels are elevated, the CRP-cAMP complex can bind to its operator and partially activate expression of the transport operon, but not the catabolic operon (Figure 8B). When both GlcN-6P and CRP-cAMP are present, an activating complex is formed with SiaR that induces expression of the two adjacent operons (Figure 8C). When the helical phase of the two operators is altered, SiaR can only regulate the *nan *operon while CRP can only regulate the *siaPT *operon (Figure 8D). Interaction between SiaR and CRP is necessary for regulation.

The interaction of CRP with another transcriptional regulator is not an unusual phenomenon, however the regulation of the adjacent *nan *and *siaPT *operons by CRP and SiaR appears to operate via a novel regulatory mechanism. What makes this regulatory region unique is that it appears that the two operons are regulated by one set of operators. Other examples of divergent operons regulated by CRP and additional regulators operate by distinctly different mechanisms. The most common mechanism is the formation of a repression loop. An example of this is in the *glp *regulon of *E. coli *[19]. As with the *siaPT *operon of NTHi, only one of the divergent *glp *operons is induced by CRP [19]. The difference between these two systems is that the repressor GlpR binds to four operators in the intergenic region and forms a repression loop [19]. The two divergent operons of the L-rhamnose catabolic regulon of *E. coli *utilize yet another mechanism. In addition to having multiple CRP binding sites, the two *rha *operons are regulated by separate transcriptional regulators, RhaR and RhaS [20]. RhaR and CRP interact to regulate the *rhaSR *operon while RhaS and CRP interact to regulate the *rhaBAD *operon [20,21].

SiaR shares functional similarity to NagC, the regulator of *N*-acetylglucosamine catabolism in *E. coli*. Like SiaR, NagC regulates the expression of *nagA *and *nagB*, as well as a number of additional genes. Also, SiaR and NagC both regulate divergently transcribed operons and CRP is involved in regulation [22,23]. There are a number of striking differences as well. GlcNAc-6P is the inducer of the NagC regulon. Just as inactivation of *nagB *causes induction of SiaR-regulated genes, the inactivation of *nagA*, and the subsequent accumulation of GlcNAc-6P, induces NagC-related genes [22]. NagC is displaced from its binding site in the presence of GlcNAc-6P [22] while SiaR appears to always be bound to its operator. In *E. coli*, the alteration of phasing between NagC operator sequences results in derepression of both divergently transcribed operons. This is due to the inability of NagC to form a repression loop that is required for NagC-mediated repression [24]. This differs significantly with what we observed in SiaR regulation. In our studies, the alteration of phasing did not result in derepression, but instead uncoupled SiaR- and CRP-mediated regulation of the *nanE *and *siaP *genes. The differences between SiaR and NagC suggest that, while some functional similarity exists between the two regulators, they both employ different mechanisms.

Given the nature of regulation by SiaR and CRP, the *nan *and *siaPT *operons will never be maximally expressed when *H. influenzae *is in its natural environment. This is due to a number of factors, including the low abundance of sialic acid in the host and the rapid utilization of intracellular sialic acid. Instead, regulation acts to subtly modulate expression of the operons, keeping expression under constant control so that catabolism does not outpace utilization and the expression of the transporter is appropriate for the availability of the ligand. These requirements are also in balance with the need to prevent the accumulation of inhibitory amounts of sialic acid, however, this need is likely minimal considering the factors of sialic acid availablity and utilization discussed above.

The role of CRP in the regulation of sialic acid transport and catabolism suggests that sialic acid is utilized as an emergency carbon source in the host. *H. influenzae *can use sialic acid as a sole carbon source as efficiently as glucose [10]. Sialic acid catabolism is not required for virulence as a *nanA *mutant exhibits increased fitness in multiple infection models [13]. However, the fact that catabolism is present and conserved among *H. influenzae *strains suggests that it provides some advantage to the organism. The previous study examining virulence of a *nanA *mutant was performed using an encapsulated, invasive type B strain rather than a non-typeable strain and did not test all possible environments within the host [13]. Additionally, intranasal mixed-challenge experiments did not reveal an advantage for either the wild-type or *nanA *mutant strain [13]. Therefore, it is possible that sialic acid catabolism is advantageous in certain conditions or has increased importance for non-typeable strains. The need for cell surface sialylation has been well established in multiple infection models for NTHi, but it is unclear at this time if and when catabolism is required.

## Conclusions

GlcN-6P, an intermediate in the catabolism of sialic acid, was found to function as a co-activator of SiaR in the regulation of the catabolic and transport operons for sialic acid in NTHi. SiaR functions as both a repressor and an activator, depending on conditions, and is required for CRP-dependent activation of the catabolic operon. Direct interactions between SiaR and CRP are likely involved in regulation.

## Methods

### Bacterial strains, media and growth

The strains used in this study are listed in Table 1. *E. coli *was grown at 37°C in Luria-Bertani (LB) medium with or without agar (2%) and supplemented with antibiotics as needed. NTHi strain 2019 [25] and derivatives thereof were used in this study. *H. influenzae *was grown at 37°C in the presence of 5% CO_2 _on brain heart infusion agar (Difco Laboratories, Detroit, MI) supplemented with 10 μg/ml hemin and 10 μg/ml β-NAD (sBHI). Kanamycin-resistant *H. influenzae *were selected on sBHI agar containing 15 μg/ml ribostamycin in the absence of additional CO_2_. Spectinomycin was added to sBHI at a concentration of 25 μg/ml. RPMI 1640 media (Sigma-Aldrich, Saint Louis, MO) was used as a sialic acid-free chemically defined media. Supplemented RPMI (sRPMI) was prepared with protoporphyrin IX (1 μg/ml), hypoxanthine (0.1 mg/ml), uracil (0.1 mg/ml), β-NAD (10 μg/ml), and sodium pyruvate (0.8 mM). Neu5Ac (100 μM) and cAMP (1 mM) were added as indicated.

**Table 1:** Strains and plasmids

Strain or plasmid	Genotype, relevant phenotype or selection marker	Source or reference
Strains		
*E. coli *DH5α		Invitrogen
E. coli BL21 Star		Invitrogen
NTHi 2019	Clinical respiratory isolate	[25]
JWJ091	NTHi 2019Δ*cyaA *mutant	This study
JWJ093	NTHi 2019Δ*cyaA *Δ*siaR *mutant, kanamycin resistant	This study
JWJ112	NTHi 2019Δ*cyaA *Δ*nanA *mutant	This study
JWJ114	NTHi 2019Δ*cyaA *Δ*nagA *mutant	This study
JWJ116	NTHi 2019Δ*cyaA *Δ*nagB *mutant	This study
JWJ118	NTHi 2019Δ*cyaA *Δ*nanK *mutant	This study
JWJ120	NTHi 2019Δ*cyaA *Δ*nanE *mutant	This study
JWJ159	NTHi 2019Δ*cyaA *mutant with 5 bp insertion between SiaR and Crp operators	This study
JWJ160	NTHi 2019Δ*cyaA *Δ*nagB *mutant with 5 bp insertion between SiaR and Crp operators	This study
Plasmids		
pGEM-T Easy	PCR-cloning vector	Promega
pGEM-T	PCR-cloning vector	Promega
pCR2.1	PCR-cloning vector	Invitrogen
pCR2.1_443	pCR2.1 with PCR fragment spanning	This study
pUC19	General cloning vector	New England Biolabs
pET-24(+)	Expression vector	Novagen
pUC19-142del	*nanA *deletion construct	This study
pUC19-142*sacB*	*nanA*::*tetR*-*sacB*/*kan*^*R*^	This study
pJJ150	*siaR *knockout vector	[14]
pJJ185	C-terminal his-tagged SiaR expression vector	[14]
pJJ260	*tetR*-*sacB*/*kan*^*R *^cassette	
pJJ276	C-terminal his-tagged CRP expression vector	This study
pJJ279	*cyaA *deletion construct	This study
pJJ290	*cyaA*::*tetR*-*sacB*/*kan*^*R*^	This study
pJJ308	*nagA *deletion construct	This study
pJJ309	*nagB *deletion construct	This study
pJJ310	*nanK *deletion construct	This study
pJJ311	*nanE *deletion construct	This study
pJJ313	*nagA*::*tetR*-*sacB*/*kan*^*R*^	This study
pJJ314	*nagB*::*tetR*-*sacB*/*kan*^*R*^	This study
pJJ315	*nanK*::*tetR*-*sacB*/*kan*^*R*^	This study
pJJ316	*nanE*::*tetR*-*sacB*/*kan*^*R*^	This study
pJJ321	pCR2.1_443 with 5 bp deletion	This study
pJJ331	pJJ321 with *tetR-sacB*/*kan*^*R*^	This study

### Construction of mutants

Genetic manipulations were performed using established techniques. Restriction enzymes, Antarctic Phosphatase, and T4 polymerase were obtained from New England Biolabs (Beverly, MA) and were used following established protocols. The Expand High Fidelity PCR System (Roche Applied Science, Indianapolis, IN) was used for PCR reactions. Oligonucleotide primers were designed and ordered from Integrated DNA Technologies (Coralville, IA) and are listed in Table 2. Plasmids were transformed and maintained in MAX Efficiency DH5α Chemically Competent cells (Invitrogen). Competent *H. influenzae *cells were prepared using the M-IV method and transformed as described previously [26]. cAMP (1 mM final concentration) was added to *cyaA *mutant strains 30 minutes prior to the addition of DNA to aid in the development of competence.

**Table 2:** Oligonucleotide primers

Oligonucleotide primer	**Sequence**^**1**^	Use
140F3	gaa ttc CTG CTT CTT CAT TAA GTT CTC GC	*nagA *upstream
140R5	ccc ggg CAT ATT CTG TTC CTA ATA TCA ACA TCA GTT	*nagA *upstream
140F6	ccc ggg TAA TAG TAA ACA CTT AAA TAG TTA ATT GAT TTA AAA ATC	*nagA *downstream
140R6	gca tgc TCA AAA ACA GCA ACA CGG TGC	*nagA *downstream
141F1	gaa ttc CAT CAT CGC TGA AAC AGG C	*nagB *upstream
141R3	ccc ggg CAT ATT AGC CTT CCT TTA TTA TTG ACC G	*nagB *upstream
141F3	ccc ggg TAG AGA TCT ATT CTT CAT CTT TAT GTA GGG	*nagB *downstream
141R4	gca tgc GGT TTC AAC GCT AGT TTG GTC G	*nagB *downstream
144F1	gaa ttc CCG TCC TTT TGT GAA TGT CC	*nanK *upstream
144F3	ccc ggg CAT AAC TTA TCC TTA TAG TGT AAA GTC TTT TCT CAC	*nanK *upstream
144F2	ccc ggg AAG CCT GAA GGA ACA ATT TAT GGC TAA	*nanK *downstream
144R2	gca tgc GCC GTT TCA GCA GAA TAA CCA G	*nanK *downstream
145F5	gaa ttc CGC TCC TGT GTG AAC TTA TG	*nanE *upstream
145R5	ccc ggg CAT ATA ACA CCC CTC ATT TAA ATC TGA AT	*nanE *upstream
145F6	ccc ggg TAG TCG TAA GAC GTG TGA GAA AAG ACT T	*nanE *downstream
145R6	gca tgc CGA ACG CAA AAT CGT ATC GGC	*nanE *downstream
604F1	gag ctc CAT TTT GCT GAC GAG GAA CTG	*cyaA *upstream
604R1	ccc ggg CAT TAC ATA AAC ACC TAA AAT TGG TGG	*cyaA *upstream
604F2	ccc ggg TAA TAT TTT CCT GTG GTT GAT AGG TTA CC	*cyaA *downstream
604R2	aag ctt AAA GCA ATG GAG TGG ACC ACA ATT	*cyaA *downstream
145R8	CCG CAG CAA TTT TTG TCC	PCR SOEing
145M2	TTT ATG AAA AAA CAC TTC AAA AAT	PCR SOEing
145M3	ATT TTT GAA GTG TTT TTT CAT AAA *TTA AA*T GTG ATC AAC TTC TC	PCR SOEing
146R2	CCA TTA CGG CAC ACT AAA GAG G	PCR SOEing
957F2	aag ctt AAT AAA ACG GAA TTT TTG AAA CAG G	CRP expression
957R2	ctc gag TCT TGC GCC ATA TAC AAC GAT TGT	CRP expression
P146F1	ACA CCC CTC ATT TAA ATC TGA ATA AAT CAC	EMSA
P146R4	CCC CCA AAA TAG GAT TCG	EMSA
145R7	CGA CAG GTT GGC AAG AAG AAA TAA GAC C	Primer extension
145R1	ATC AGC GGC AAG AAC AGC AG	Primer extension

^1^Restriction sites added to aid in subcloning are indicated by lowercase letters. Bases added for 5 bp insertion are italicized.

All mutants used in this study were constructed as non-polar deletions using a counter-selectable cassette. The cassette used was a variation of *sacB *cassettes that have been described previously [27,28]. In this cassette, which is described in more detail in work to be submitted elsewhere, *sacB *is under the control of the tetracycline promoter and Tet repressor. The cassette also contains genes for the repressor, *tetR*, and *nptII*, a kanamycin resistance marker. This allows for inducible expression of *sacB *in the presence of the tetracycline analog chlortetracycline. Constructs for mutagenesis were prepared for each gene using the design detailed as follows. Regions flanking the target gene were amplified by PCR using primers that had restriction sites added to the 5'-end. These primers were designed to contain the start codon for the upstream fragment and stop codon for the downstream fragment. These products were cloned into pGEM-T (Promega, Madison, WI) and sequentially subcloned into pUC19 using the primer-encoded restriction sites. The resulting plasmid contained the flanking regions ligated to form an open reading frame consisting of a start codon, SmaI site, and stop codon. This plasmid would serve as the deletion construct. SmaI was then used to open the plasmid and the *sacB*-Kan^R ^cassette was inserted. The resulting plasmid was transformed into the desired NTHi strain, selecting for resistance to ribostamycin. A Rib^R^, Suc^S ^isolate was then transformed with the deletion construct and transformants were selected on LB agar supplemented with 5% sucrose, chlortetracycline (1 μg/ml), hemin, and NAD. Deletions were confirmed by PCR. Confirmed mutants were then able to be transformed with the *sacB*-Kan^R ^cassette to delete additional genes.

### PCR SOEing and mutagenesis

PCR splicing by overlap extension (PCR SOEing) was used to insert 5 bp between SiaR and CRP operators. Primers were designed to insert 5 bp between the operators of SiaR and CRP while conserving the 3 bp that are shared between the two (Table 2). The junction primers contained a 24 bp overlap to allow for splicing. Fragments were amplified by PCR with primer pairs 145R8/145M2 and 145M3/146R2 and products were purified using the QiaQuick PCR Clean Up Kit (Qiagen). PCR products were quantified with NanoDrop and mixed to yield a final concentration of 5 ng/μl of each and this mixture was used as the template in the SOEing reaction with primers 145R8 and 146R2. The product from the splicing reaction was cleaned up and used for transformation.

Transformation of NTHi strains was performed as detailed above. JWJ091 and JWJ116 were transformed with the plasmid pJJ331, a construct that spans from within the *nan *operon and into the *siaPT *operon and has the *sacB*-Kan^R ^cassette inserted near the insertion target. pJJ331 had an unintentional mutation in the CRP binding site that allowed for the plasmid to be maintained in *E. coli*. Kan^R^, Suc^S ^transformants were then transformed with the PCR SOEing product and selected for growth on sucrose. Transformants were then screened by PCR and sequenced to confirm the presence of the 5 bp insertion and the absence of additional mutations. The resultant strains, JWJ159 (2019*cyaA*+5 bp) and JWJ160 (2019*cyaAnagB*+5 bp) were used for subsequent analysis.

### RNA extraction and transcriptional analysis

RNA was extracted using the hot acid phenol method as described previously [29]. DNA was removed from extracted RNA by digestion with DNase I (New England Biolabs) and cleaned up with the RNeasy Mini Kit (Qiagen, Valencia, CA). RNA quality was assessed with an Agilent 2100 Bioanalyzer (Agilent, Santa Clara, CA) and the concentration was determined using a NanoDrop ND-1000 Spectrophotometer (NanoDrop Technologies, Wilmington, DE). For real time RT-PCR analysis, primer/probe sets were obtained using the Custom TaqMan Gene Expression Service (Applied Biosystems, Foster City, CA). Primer/probe sets were designed using the sequence of HI0145 and HI0146 from *H. influenzae *2019. A primer/probe set for the 16S rRNA of *H. influenzae *was designed and used as a control. The TaqMan RNA-To-C_T _*1-Step *Kit (Applied Biosystems) was used following the manufacturer's protocol. Reactions were set up in triplicate using 20 ng of RNA. Reactions were carried out using the StepOnePlus Real Time PCR System (Applied Biosystems) with StepOne analysis software. Results were calculated using the comparative C_T _method to determine the relative expression ratio between RNA samples. The primer and probe set for HI16S rRNA was used as the endogenous reference to normalize the results. Two independent sets of RNA samples were used for each experiment and the mean fold change is reported. Data are expressed as mean +/- SD.

### Protein expression and purification

SiaR was expressed and purified as described previously [14], with modified buffers to enhance stability of the purified protein and an additional purification step. Cells were resuspended in the SiaR lysis and equilibration buffer (10 mM Tris, pH 8.0, 300 mM NaCl, 0.1% CHAPS) prior to lysis by French press. After protein binding, the resin was washed with the SiaR wash buffer (10 mM Tris, pH 8.0, 1,150 mM NaCl, 10% glycerol, 0.1% CHAPS, 5 mM imidazole) and protein was eluted with the SiaR elution buffer (10 mM Tris, pH 8.0, 150 mM NaCl, 10% glycerol, 0.1% CHAPS, 500 mM imidazole). The purified protein was concentrated using an Amicon Ultra centrifugation filter (Millipore, Billerica, MA) with a 10 kDa molecular weight cutoff. The protein sample was then desalted into the SiaR storage buffer (10 mM Tris, pH 8.0, 150 mM NaCl, 10% glycerol, 0.1% CHAPS) using FPLC through a 10 ml (2-5 ml) HiTrap Desalting Column (GE Healthcare, Piscataway, NJ). Protein concentration was determined using the NanoDrop ND-1000 Spectrophotometer and an extinction coefficient of 7,575 M^-1 ^cm^-1^. Aliquots of the purified protein were frozen in liquid nitrogen and stored at -80°C until needed.

CRP was expressed and purified in a similar manner. Primers were used to amplify *crp *with the restriction sites HindIII and XhoI on the 5' and 3' ends, respectively (Table 2). The 41 bases immediately upstream of *crp *were included to ensure that the native bacterial translation signals were present. The downstream primer included the last codon of the *crp *open reading frame, excluding the stop codon, to allow for the fusion of a multiple-histidine tag. The PCR product was cloned into pGEM-T and subsequently subcloned into pET-24(+) (Novagen, Madison, WI) using the HindIII and XhoI sites. The resulting plasmid, pJJ276, was expected to express CRP with a carboxy-terminal His•Tag.

Protein expression was induced using the Overnight Express Autoinduction System 1 (Novagen) grown at 37°C overnight. Expressed protein was purified using the BD TALON Metal Affinity Resin (BD Biosciences, Palo Alto, CA). Purification was performed in native conditions following the manufacturer's protocol and using the suggested TALON buffers. Eluted fractions were examined by SDS-PAGE and fractions containing CRP were pooled. Protein was concentrated using an Amicon Ultra centrifugation filter and desalted as described above. The protein concentration was determined using the NanoDrop ND-1000 Spectrophotometer and an extinction coefficient of 21,555 M^-1 ^cm^-1^. Purified protein was stored at 4°C.

### Electrophoretic mobility shift assay

Electrophoretic mobility shift assay (EMSA) was used to study the binding of SiaR and CRP to potential promoter sequences as done previously [14]. The probe for EMSA was amplified by PCR using primer pairs P146F1 and P146R4 (Table 2), resulting in a probe that spans the region from the *nanE *start codon to +18 of the *siaPT *transcript. Binding reactions were prepared using the EMSA Kit (Molecular Probes, Eugene, OR) following the manufacturer's directions with some modifications. Binding reactions consisted of the binding buffer (150 mM KCl, 0.1 mM DTT, 0.1 mM EDTA, 10 mM Tris, pH 7.4), the DNA probe (15 nM), and 1 μM SiaR and/or CRP. Control reactions without protein were set up for each probe. Reactions were incubated at room temperature for 20 minutes. After incubation, 6× EMSA gel-loading solution was added and reactions were loaded onto a 6% DNA Retardation Gel (Invitrogen) with prechilled 0.5× TBE buffer and run at 200 V for 60 minutes. After electrophoresis, the gel was stained with SYBR Green EMSA gel stain and bands were visualized by UV transillumination. Images were captured using a Kodak EDAS 120 camera with an EDAS 590 mm filter (Eastman Kodak Company, Rochester, NY). cAMP was added to reactions when indicated to a final concentration of 100 μM.

### Primer extension analysis

Primer extension analysis was used to identify the transcriptional start sites for both *nan *and *siaPT *operons. Primers 145R7 (*nan*) and 146R1 (*siaPT*) were labeled with ^32^P using T4 polynucleotide kinase (New England Biolabs) and γ-[^32^P]-ATP (GE Healthcare). Illustra Microspin G-25 columns (GE Healthcare) were used to remove unincorporated ^32^P. The primer extension reaction was performed using SuperScript III First-Strand Synthesis SuperMix (Invitrogen) following the supplied protocol. After first-strand synthesis RNA was degraded by incubation with RNase A (New England Biolabs) at 37°C for 15 min. Nucleic acids were precipitated by the addition of 300 μl of chilled ethanol, incubation in a dry ice bath for 15 min, and centrifugation at 4°C. Dried samples were dissolved in loading buffer (98% deionized formamide, 10 mM EDTA, 0.025% xylene cyanol FF, 0.025% bromophenol blue) prior to loading on sequencing gel. Sequencing reactions were set up for each labeled primer using the SequiTherm EXCEL II DNA Sequencing Kit (Epicentre Technologies, Madison, WI). A PCR fragment amplified with the primers 145R7 and 146R1 was used as a template. Sequencing and primer extension reactions were loaded onto an 8% sequencing gel. After electrophoresis, the gel was dried and exposed to film at -80°C.

### NMR spectroscopy

Strains 2019 wild-type and the 2019Δ*cyaA *Δ*nagB *mutant were grown in 100 ml cultures of sRPMI without Neu5Ac to early exponential phase. Neu5Ac, cAMP, or both were added and cultures were incubated for 20 min. Cells were pelleted and resuspended in 0.5 ml of MOPS buffer (40 mM MOPS, pH 7.3, with 50 μl D_2_O). Phosphorus NMR spectra were acquired at 162 MHz on a 400 MHz Varian Inova spectrometer in a 5 mm probe. Spectra were obtained upon excitation with at 45° pulse and digitization of 0.8 s followed by a delay of 1.7 s for recovery between scans. Spectra 20 kHz wide were collected and processed with gaussian line-broadening of 0.1 s prior to Fourier transformation. Samples were maintained at 15°C, 2048 transients were averaged in an experiment lasting 1.5 hours. For each sample, two such spectra were collected one after the other. These were not significantly different, indicating that relatively minor changes take place on the time scale of data collection. However a third spectrum collected some 13 hours later indicated significant change in some cases. Chemical shifts were referenced relative to external 85% phosphoric acid at 0 ppm.

## Authors' contributions

JWJ constructed all mutant strains, designed and carried out the transcriptional analysis, performed the EMSA analysis, and wrote the primary draft of the manuscript. HS and AFM performed the NMR studies and assisted in data analysis. MAA assisted in the conception of the study and contributed to data analysis and manuscript editing. All authors read and approved the final manuscript.
